# Towards Accurate Identification of Antibiotic-Resistant Pathogens through the Ensemble of Multiple Preprocessing Methods Based on MALDI-TOF Spectra

**DOI:** 10.3390/ijms24020998

**Published:** 2023-01-05

**Authors:** Chia-Ru Chung, Hsin-Yao Wang, Po-Han Chou, Li-Ching Wu, Jang-Jih Lu, Jorng-Tzong Horng, Tzong-Yi Lee

**Affiliations:** 1 Kobilka Institute of Innovative Drug Discovery, School of Medicine, The Chinese University of Hong Kong, Shenzhen 518172, China; 2 School of Life Sciences, University of Science and Technology of China, Hefei 230026, China; 3 Department of Laboratory Medicine, Chang Gung Memorial Hospital at Linkou, Taoyuan 333423, Taiwan; 4 Ph.D. Program in Biomedical Engineering, Chang Gung University, Taoyuan 333323, Taiwan; 5 Department of Computer Science and Information Engineering, National Central University, Taoyuan 320317, Taiwan; 6 Department of Biomedical Sciences and Engineering, National Central University, Taoyuan 320317, Taiwan; 7 Research Center for Emerging Viral Infections, Chang Gung University, Taoyuan 333323, Taiwan; 8 College of Medicine, Chang Gung University, Taoyuan 333323, Taiwan; 9 Department of Medical Biotechnology and Laboratory Science, Chang Gung University, Taoyuan 333323, Taiwan; 10 Department of Bioinformatics and Medical Engineering, Asia University, Taichung 41354, Taiwan; 11 Warshel Institute for Computational Biology, School of Life and Health Sciences, The Chinese University of Hong Kong, Shenzhen 518172, China; 12 Institute of Bioinformatics and Systems Biology, National Yang Ming Chiao Tung University, Hsinchu 300093, Taiwan

**Keywords:** MALDI-TOF MS, machine learning, antibiotic resistance

## Abstract

Matrix-assisted laser desorption/ionization time-of-flight (MALDI-TOF) mass spectrometry (MS) has been used to identify microorganisms and predict antibiotic resistance. The preprocessing method for the MS spectrum is key to extracting critical information from complicated MS spectral data. Different preprocessing methods yield different data, and the optimal approach is unclear. In this study, we adopted an ensemble of multiple preprocessing methods––FlexAnalysis, MALDIquant, and continuous wavelet transform-based methods––to detect peaks and build machine learning classifiers, including logistic regressions, naïve Bayes classifiers, random forests, and a support vector machine. The aim was to identify antibiotic resistance in *Acinetobacter baumannii*, *Acinetobacter nosocomialis*, *Enterococcus faecium*, and Group B *Streptococci* (GBS) based on MALDI-TOF MS spectra collected from two branches of a referral tertiary medical center. The ensemble method was compared with the individual methods. Random forest models built with the data preprocessed by the ensemble method outperformed individual preprocessing methods and achieved the highest accuracy, with values of 84.37% (*A. baumannii*), 90.96% (*A. nosocomialis*), 78.54% (*E. faecium*), and 70.12% (GBS) on independent testing datasets. Through feature selection, important peaks related to antibiotic resistance could be detected from integrated information. The prediction model can provide an opinion for clinicians. The discriminative peaks enabling better prediction performance can provide a reference for further investigation of the resistance mechanism.

## 1. Introduction

Antibiotic resistance can be defined as the ability of infection-causing bacteria to resist the actions of drugs that have previously been successfully used. The rapid increase and spread of antibiotic-resistant pathogenic bacteria have become a serious public health concern. Generally, antibiotic susceptibility testing (AST) is performed to identify antibiotics effective in treating bacterial infections. The commonly used AST test methods in clinical microbiology laboratories, include paper disc, and mini-broth, macro-broth, and agar dilutions. These methods, have high sensitivity and specificity. However, they are time-consuming, yield results late and, in some cases, may pose a threat to the patients [1]. Therefore, it is necessary to implement other methods to rapidly detect antibiotic resistance in routine microbiological laboratories.

Matrix-assisted laser desorption/ionization time-of-flight (MALDI-TOF) mass spectrometry (MS) is a rapid and cost-effective method widely used in clinical microbiology laboratories. It is typically used in the identification of bacterial species. However, recently, it has been applied for the detection of antibiotic resistance [2,3,4]. Each microorganism has a unique mass spectral fingerprint, allowing MALDI-TOF MS to be used for species identification, antibiotic resistance detection, and strain typing [5,6,7,8,9,10]. The mass spectrum of a sample can be regarded as a pattern that represents the distribution of ions using the mass-to-charge ratio (*m*/*z*). Theoretically, the signals generated by MALDI-TOF MS correspond to ionized protein or peptide fragments. However, a mass spectrum contains peaks representing ionized protein or peptide fragments, and baseline drift and background noise caused by matrix impurities and electronic interference. Consequently, signal preprocessing of MS data, including smoothing, removing baseline, and identifying peaks as biomarkers from a mass spectrum, is a crucial step [11].

Various MS processing tools and methods have been developed to extract information that can be used for further analysis. An optional tool is the commercial software provided by mass spectrometer manufacturers. Commercial software can help make internal adjustments and specific corrections to the mass spectra based on the basic settings of the mass spectrometer. Many open-source software packages, such as the MALDIquant package for R and PLS Toolbox software for MATLAB, are also available; these employ several data preprocessing approaches and offer easy-to-use functions to find the peaks that highly affected the performance of antibiotic resistance classification and species analysis [12,13]. A continuous wavelet transform (CWT)-based method is available for identifying peaks by transforming a spectrum into a matrix of wavelet coefficients, which can provide additional information [14,15]. An early study showed that the CWT-based method performed well in detecting true peaks [16]. Furthermore, some methods convert spectral signals into wavelet coefficients or calculate the statistics of spectral signals as features, rather than using peaks as features for cancer classification [17,18].

The set of peaks after signal preprocessing had a significant influence on the entire study. In recent years, several studies have been conducted on the identification of antibiotic resistance using MALDI-TOF MS [4,9,10,19,20,21,22,23]. These studies chose a proper preprocessing method to detect peaks and analyze the information from the spectra. Wang et al. [19] used FlexAnalysis software (Bruker Daltonics) to process MS data, and trained machine learning algorithms to rapidly detect heterogeneous vancomycin-intermediate *Staphylococcus aureus*. Tang et al. [20] used the MALDIquant package to detect and establish reference peaks. Similarly, machine learning models were used to classify methicillin-susceptible *S. aureus* (MSSA) and methicillin-resistant *S. aureus* (MRSA) and to verify the important peaks. Huang et al. [21] detected peaks and aligned the MALDI-TOF spectra using the Mass-Up software. Subsequently, five machine learning models were built to identify carbapenem-resistant *Klebsiella pneumoniae* strains.

Several studies have confirmed that MALDI-TOF MS is a good method for solving bacteria-related problems [4,9,10,19,20,21,22,23]. In antibiotic resistance-related studies, the combination of MS and machine learning methods appears to be popular and useful for the detection of antibiotic resistance. Different preprocessing methods can detect different peaks even from the same MS dataset. Identifying the discriminative peaks which highly affected the performance of prediction model can promote the identification of antibiotic resistance using MALDI-TOF MS. In this study, we adopted an ensemble of multiple data preprocessing methods to process MALDI-TOF MS data and built machine learning models to identify antibiotic resistance.

## 2. Results

### 2.1. Peak Counts

**Figure 1:**
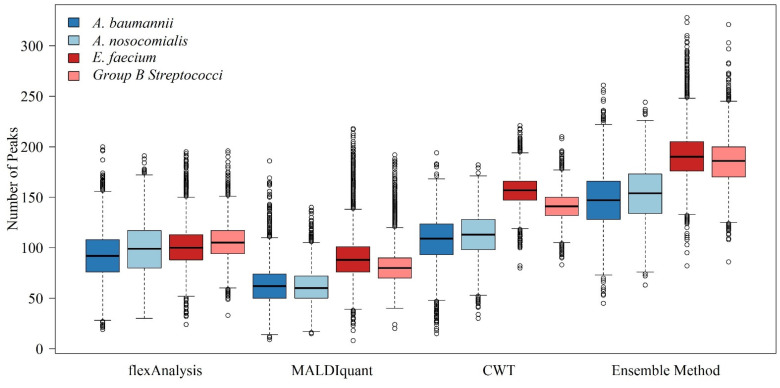
Boxplots of the numbers of peaks with different preprocessing methods for *A. baumannii*, *A. nosocomialis*, *E. faecium*, and Group B *Streptococci*.

We counted the number of peaks in each spectrum using different preprocessing methods. Figure 1 shows a comparison of the different preprocessing methods for each bacterial species. The ensemble method represents the proposed combined multiple preprocessing method. Regardless of the type of bacteria, the number of informative peaks extracted using the combined method was greater than the other individual methods. This implies that additional information can be used.

### 2.2. Distribution of Benchmark Peaks

**Figure 2:**
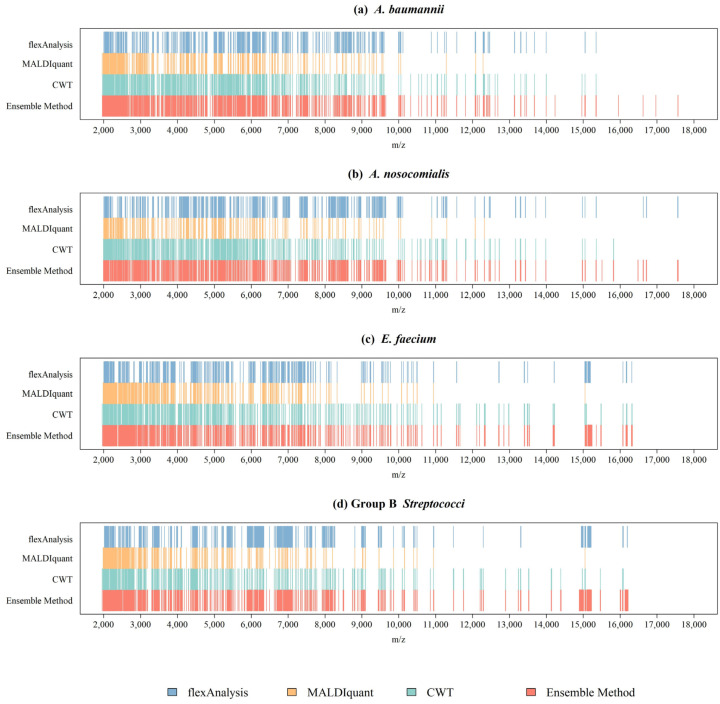
Distributions of benchmark peaks with different preprocessing methods for (**a**) *A. baumannii*, (**b**) *A. nosocomialis*, (**c**) *E. faecium*, and (**d**) Group B *Streptococci*.

Figure 2a shows a plot of the benchmark peak distribution for each preprocessing method for *A. nosocomialis*. The *x*-axis was *m*/*z*. We listed the *m*/*z* values of all benchmark peaks and drew straight lines at these positions to generate a graph to observe the difference in peaks between the different preprocessing methods. Different methods exhibit obvious differences at certain intervals. The combined method integrates all the information. Therefore, the benchmark peaks obtained using the combined method were the highest, and almost covered the peaks of the other methods. *A. baumannii*, *E. faecium*, and GBS are shown in Figure 2b–d respectively. The number of benchmark peaks was 679 for *A. nosocomialis*, 676 for *A. baumannii*, 657 for *E. faecium*, and 638 for GBS. These peaks were used to construct machine learning classifiers to identify antibiotic resistance.

### 2.3. Performances of Models

**Figure 3:**
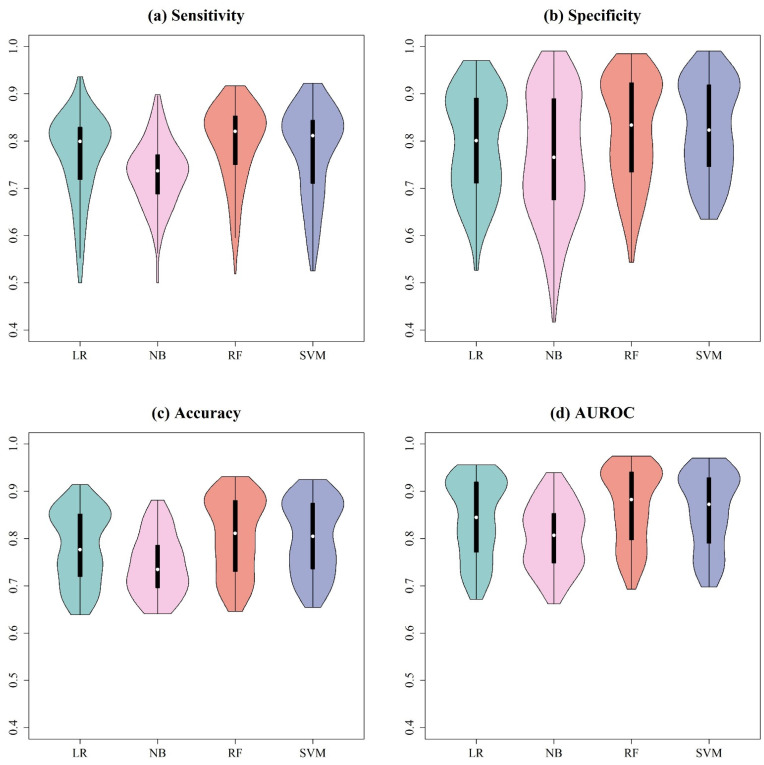
Violin plots for (**a**) sensitivity, (**b**) specificity, (**c**) accuracy, and (**d**) AUROC using different machine learning classification algorithms. The different preprocessing methods were integrated.

To determine the most suitable machine learning classification algorithms for prediction, LR, NB, RF, and SVM were first constructed using the peaks extracted by different signal preprocessing methods. Supplementary Tables S1–S4 show the means and standard deviations of the 10-fold cross-validation on training datasets for each evaluation metrics with all combinations of preprocessing methods and machine learning models for *A. baumannii*, *A. nosocomialis*, *E. faecium*, and GBS, respectively. In addition, Figure 3 shows violin plots for different machine learning classification algorithms. Regardless of the preprocessing method used, the RF models performed better than other models. Therefore, the RF model was the final model for feature selection.

### 2.4. Feature Selection on RF

**Figure 4:**
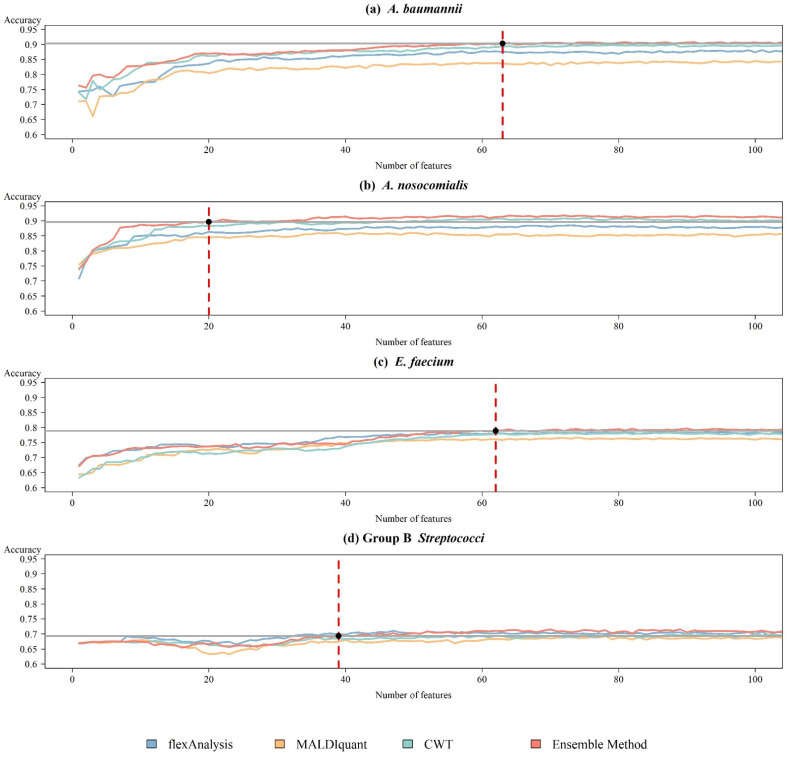
Trend plots of the accuracy using ensemble method preprocessing and RF during the feature selection process on the training datasets for (**a**) *A. baumannii*, (**b**) *A. nosocomialis*, (**c**) *E. faecium*, and (**d**) Group B *Streptococci*. The red dotted line indicated the number of features that were selected.

After determining the best prediction model, we applied ensemble-based feature selection to select the influential features and rebuilt the RF model by adding features based on the ranked importance. Except for the combined method, the features extracted by each individual preprocessing method were selected using the same procedure. Figure 4a shows the mean accuracy of 10-fold cross-validation when selecting different numbers of features for *A. nosocomialis*. For the combined method, the model achieved 89.84% accuracy, similar to the use of all the features when selecting 20 features. Figure 4b shows the results for *A. baumannii*, which had an accuracy of 90.41% with 63 features. Figure 4c shows the results for *E. faecium*, which required 62 features to achieve a similar accuracy of 79.02%. Figure 4d shows the result for GBS, which required 39 features to obtain a similar accuracy of 69.53%. The model using the combined method yielded a better performance with fewer features. Table 1 presents the details of each bacterium. When using the individual preprocessing method, the model required more features to achieve a performance similar to that of the model using the ensemble method, or could not even be achieved in *A. nosocomialis*, *A. baumannii*, and *E. faecium*. Although flexAnalysis achieved better accuracy when using fewer features in Group B *Streptococci*, the combined method still yielded a stable performance if the number of features was increased. Table 2 shows the performance of the feature selection in the independent test.

Subsequently, we observed important peaks of the combined method. Supplementary Table S5 shows the frequency of occurrence of discriminative peaks in *A. nosocomialis*. The top five peaks—2949, 4181, 8549 3910, and 8559 *m*/*z*—were detected using all preprocessing methods. However, the peaks at 5417, 7796, 5425, and 5502 *m*/*z* were detected using the CWT-based method more often, and peaks at 7003 and 4280 *m*/*z* were found using flexAnalysis. The informative peaks of *A. baumannii* are shown in Supplementary Table S6. Almost all the top important peaks, such as those at 6160, 9072, 12,320, and 6150 *m*/*z*, had a similar frequency of occurrence in at least two preprocessing methods, except for 7853, 7319, and 3927 *m*/*z*, which were easier to find by the CWT-based method. Supplementary Table S7 presents the conditions for *E. faecium*. The peaks at 6689, 6602, and 3302 *m*/*z* were detected using three different methods. However, the proportion of occurrence differed. Although the peaks at 6356 and 6360 *m*/*z* were detected by the MALDIquant and CWT-based method, respectively, information regarding these two peaks was almost provided by flexAnalysis software. Supplementary Table S8 shows the frequency of peak occurrence in GBS. The difference between the different methods was not obvious. The important peaks, that is, the ones at 6250, 3124, 3368, 7638, and 3820 *m*/*z*, were found using all the methods. The proportions of all were almost the same, except for those at 6288, 9797, and 4883 *m*/*z*. The peak at 6288 *m*/*z* was easier to detect using flexAnalysis, and the peaks at *m*/*z* 9797 and 4883 were extracted using the CWT-based method. Supplementary Figures S2–S5 demonstrate typical actual measurement data. Specifically, we randomly sampled 20 resistant and 20 susceptible isolates of each species. Subsequently, the MALDI-TOF MS profiles of different isolates and the peaks that significantly affected the performance of the prediction model were illustrated. Since we shifted the MS peaks in the first step of the alignment, several peaks would not be at the exact *m*/*z* in the original MS.

## 3. Discussion

Data preprocessing is a crucial step in the analysis of mass spectra. In our study, we combined three typical preprocessing methods—FlexAnalysis, MALDIquant, and the CWT-based method—to process MALDI-TOF MS data of four bacteria acquired from Linkou CGMH, Linkou Branch and Kaohsiung Branch. We then proposed a two-stage alignment method to solve the peak shifting problem and used ensemble-based feature selection to select informative peaks. After feature extraction, we constructed a machine learning model to identify antibiotic resistance. The accuracy of RFs on the 10-fold cross-validation was 89.94% with 20 features for *A. nosocomialis*, 90.41% with 63 features for *A. baumannii*, 79.02% with 62 features for *E. faecium*, and 69.53% with 39 features for GBS. The accuracy of the independent test was 90.96% for *A. nosocomialis*, 84.37% for *A. baumannii*, 78.54% for *E. faecium*, and 70.12% for GBS.

Different spectral preprocessing methods have a significant impact on the peak (feature) number used in the machine learning model and subsequently on the model performance. Among the three preprocessing methods, the CWT method detected the most peaks, while MALDIquant detected the least peaks from the MS spectra (Figure 1). Regarding the distributions of the peaks in Figure 2, a relatively even peak distribution can be found using flexAnalysis and CWT. Peaks with *m*/*z* larger than 10,000 were still detected using flexAnalysis and CWT. In contrast, peaks detected by MALDIquant were generally distributed at *m*/*z* lower than 10,000. Moreover, Figure 2 also demonstrates that peaks were intensively detected in the range between *m*/*z* 2000 and 4000 by using MALDIquant as the spectral preprocessing method. In terms of the predictive accuracy shown in Figure 3, the model performance based on MALDIquant attained the lowest accuracy in the four study cases. Generally, the predictive accuracy based on MALDIquant was approximately 5% lower than that of the other preprocessing methods. The inferior performance could result from the fewer detected peaks in the range higher than *m*/*z* 4000. In contrast, the MALDIquant-based predictive model attained approximately 90% performance compared to the ensemble method-based model (e.g., in Figure 3a, MALDIquant-based:0.85; ensemble method-based:0.92). These results imply that spectral data ranging between *m*/*z* 2000 and 4000 contributes more than higher *m*/*z* ions in detecting antibiotic resistance. Thus, peptides ranging between *m*/*z* 2000 and 4000 would be high priority peptides for identification by proteomic tools because of their high possibility of delivering antibiotic resistance [2].

**Figure 5:**
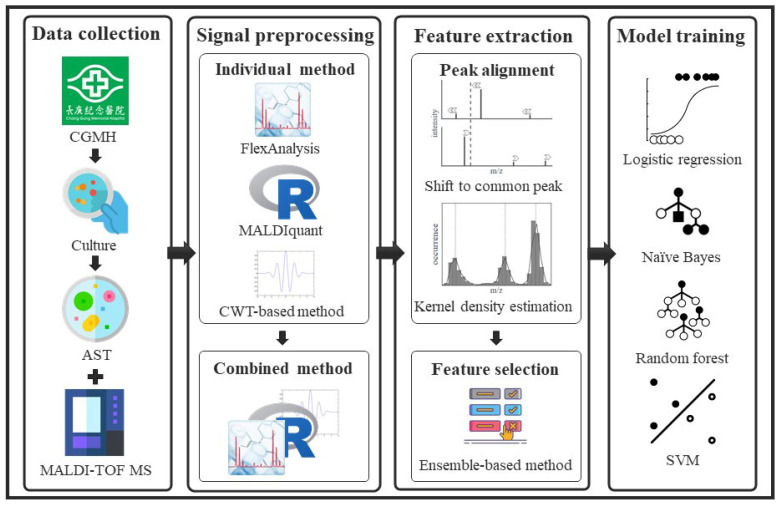
Flowchart of the study. Clinical strains were cultured for sample collection. Mass spectra and antibiotic resistance labels of the bacterial strains were obtained using MALDI-TOF MS and AST. In signal preprocessing, we considered the individual preprocessing methods and ensembled them to extract informative peaks from the raw mass spectra. In feature extraction, a two-stage alignment method was proposed to deal with the shifting problem of peaks and to convert them to available features. Feature selection was performed to identify important features of antibiotic resistance. In the model training, we constructed classification models using various machine learning algorithms.

High feature numbers are associated with overfitting of models to classifications. According to the curse of high dimensionality, higher feature numbers detected by the ensemble method than by other preprocessing methods would be susceptible to overfitting and inferior predictive performance, as shown in Figure 1. However, we observed the opposite results in which the highest performance was associated with the preprocessing method that detected the most peaks (i.e., ensemble method), while the lowest performance was associated with the preprocessing method that detected the least peaks (i.e., MALDIquant method) (Figure 4). The observations were non-intuitive and complicated, and can be attributed to several reasons. First, the number of features detected by either preprocessing method is far from the number that causes overfitting. The number shown in Figure 1 detected by the combined method was approximately 200. The difference in the number of features obtained by different preprocessing methods was approximately 100. Given the large number of bacterial isolates, the sample-feature ratio was larger than 10 for both preprocessing methods. Such a sample-feature ratio is not prone to overfitting. Second, in the workflow shown in Figure 5 we designed an RF-based feature selection prior to training using machine learning algorithms. Only features with high importance are used in machine learning models, whereas other features are filtered out. The number of features decreases significantly in this step. In the four study cases, 20 to 63 discriminative features were used in the models. Typically, such a feature number does not cause overfitting.

## 4. Materials and Methods

This study was conducted in four parts: data collection, data preprocessing, feature extraction, and model training. Clinical isolates were collected and cultured for species identification and AST. The mass spectra and antibiotic resistance labels of the bacterial isolates were obtained using MALDI-TOF MS and AST, respectively. In signal preprocessing, we considered individual preprocessing methods and combined them to extract informative peaks from the raw mass spectra. In feature extraction, a two-stage alignment method was proposed to deal with the shifting problem of peaks and convert them into available features. Feature selection was performed to identify the important features of antibiotic resistance. In model training, we constructed classification models using various machine learning algorithms. A flowchart of this study is presented in Figure 5.

### 4.1. Bacterial Isolates

All isolates analyzed in this study were collected from the Chang Gung Memorial Hospital (CGMH), Linkou Branch, and Kaohsiung Branch, Taiwan. To conduct a proof-of-concept study addressing the impact of MS data preprocessing on AST prediction, four bacterial species were used: *Acinetobacter baumannii*, *Acinetobacter nosocomialis*, *Enterococcus faecium*, and Group B *Streptococci* (GBS). It should be noted that both gram-positive bacteria (i.e., *E. faecium* and GBS) and gram-negative bacteria (i.e., *A. baumannii* and *A. nosocomialis*) were included to further investigate this generalization. Clinical isolates from the Linkou Branch were used as the training dataset, whereas those from the Kaohsiung Branch were used as the independent testing dataset.

Bacterial specimens were collected from the patients and cultured for MALDI-TOF MS and AST. The antibiotics used for each bacterium in AST were ciprofloxacin (CIP) for *A. nosocomialis* and *A. baumannii*, vancomycin (VA) for *E. faecium*, and clindamycin (CC) for GBS. These antibiotics are widely used to treat these four bacteria. However, the resistant-versus-susceptible ratios for these antibiotics were all approximately 1, which means that the clinicians only have an approximately 50% chance to use the appropriate antibiotics based on empirical therapy. Thus, an accuracy of 50% should be improved to assist in the timely use of appropriate antibiotics. More specifically, the broth micro-dilution method (BMD) was adopted to test the minimal inhibitory concentration (MIC) of CIP and CC for *Acinetobacter* spp. and GBS strains isolated from sterile specimens (i.e., blood, cerebrospinal fluid, pleural effusion, pericardial effusion, ascites, and synovial fluid). BMD was measured using a Phoenix™ M50 (Becton, Dickinson and Company, East Rutherford, NJ, USA). For *Acinetobacter* spp. and GBS strains from nonsterile specimens, the paper disc method was used to determine the AST (Creative Media Plate, New Taipei City, Taiwan). For *E. faecium*, the MIC of VA was determined by E-test (bioMérieux, Marcy-l’Étoile, France) for strains isolated from sterile specimens, whereas paper disc method was used for strains from nonsterile specimens. All AST procedures were performed according to the manufacturer’s instructions and aligned with the Clinical and Laboratory Standards Institute (CLSI) M100 guidance. AST results were classified as: resistant, susceptible, and intermediate. Intermediate isolates were classified as resistant isolates so that renders binary classifications. Table 3 shows the antibiotic resistance statistics for each bacterium in the training and independent testing datasets.

### 4.2. MALDI-TOF MS

MALDI-TOF MS was performed to generate mass spectral data and identify bacterial species using a Microflex LT mass spectrometer (Bruker Daltonik GmbH, Bremen, Germany). The experimental procedures were performed in accordance with the manufacturer’s instructions. Single colonies were selected for species identification. Formic acid (70%. Bruker Daltonik GmbH, Bremen, Germany) was used for bacterial lysis, followed by a matrix solution comprised of 50% acetonitrile containing 1% α-cyano-4-hydroxycinnamic acid, and 2.5% trifluoroacetic acid (Bruker Daltonik GmbH, Bremen, Germany). A linear positive model was used for the data acquisition. The accelerating voltage was set to +20 kV, and the frequency of the nitrogen laser was set to 60 Hz. Each sample was shot 240 times for measurement. Bacterial species were identified by MALDI-TOF MS using a Microflex LT mass spectrometer (Bruker Daltonik GmbH, Bremen, Germany). A log score generated using Biotyper 3.1 higher than 2 was used to confirm the bacterial species. We adopted the manufacturer’s instructions to conduct the MALDI-TOF analyses.

### 4.3. Proposed Signal Preprocessing Method

MsConvert, a data conversion tool provided by ProteoWizard, converts MS files into readable file formats [24]. Data preprocessing extracts discriminative peaks from the raw spectra into a peak list that includes the associated *m*/*z* and intensity for each isolate. We used three characteristic preprocessing methods and combined them in the present study.

The first method was flexAnalysis 3.3 (Bruker Daltonik GmbH, Bremen, Germany). It was used to process the MS data. Spectral signals were smoothed using the Savitzky–Golay algorithm, and baselines were removed using the top-hat method. A centroid peak detection algorithm was used for peak detection. The related parameters were signal-to-noise ratio (SNR), 2; relative and minimum intensity threshold, 0; maximum number of peaks, 200; peak width, 6; and height, 80%. After this process, peaks in the spectral range of 2000–20,000 *m*/*z* were acquired.

The second method was MALDIquant (version 1.19.3). This is an R (version 3.6.2) package used for MS analysis [12]. It provides the function “readBrukerFlexFile” which enables directly reads of MS data in the Bruker Daltonics XMASS format. During the processing stage, we used the function “removeBaseline” to correct the baseline through the top-hat method and then used the function “detectPeaks” to look for peaks. The local maxima in the spectral signals were regarded as peak candidates. These candidates were recognized as true peaks based on the full width at half maximum set to 20 and SNR set to 5. We set the median absolute deviation as the noise estimate function. The peaks had to be the highest in the given window and the SNR of the peaks had to be higher than the given threshold. The reason for the selecting the parameters was to extract peaks that could be similar to those derived from flexAnalysis.

The third method applies a CWT-based method to convert spectral signals into wavelet coefficients to obtain additional information in the wavelet space. The CWT function is expressed as follows:

$$C_{w}\left(a,\;b\right)=\frac{1}{\sqrt{a}}\int_{R}S\left(t\right)\Psi \left(\frac{t-b}{a}\right)dt$$

where *S*(*t*) is the spectral signal, *a* is the scale value, *b* is the translation value, Ψ is the mother wavelet, and *C_w_* is the 2-dimensional matrix, the row and column of which represent the scale and translation, respectively. Wavelet coefficients in the matrix represent the fitness between the spectral signal and mother wavelet. In the method, the local maxima in each row are marked and connected across adjacent rows to form ridge lines. True peaks are identified based on three rules: the scale corresponding to the maximal coefficient on the ridge line, SNR must be larger than a given threshold, and length of ridge lines should be higher than a certain threshold. In this study, we used the function “find_peaks_cwt” in the SciPy package to implement the CWT-based method [25]. More specifically, the *m*/*z* values of the MS spectrum formed a one-dimensional array to be the input of the function “find_peaks_cwt”. The parameters of the function were set as follows: widths were 1–30, the mother wavelet was the Ricker wavelet, the minimum length of the ridge line was one-quarter that the width, and the minimum SNR was 3. Similarly, the reason for the choice of the parameters was to extract peaks that could be similar to those derived from flexAnalysis. 

The three methods were combined into the ensemble method. Using different signal preprocessing methods, different peak lists were obtained from the mass spectra. To integrate the information between different methods, we collected peaks as long as they were detected by any method to form a new peak list. The positions of the same peaks detected using different methods may be slightly different. This led to peaks that might have been duplicated in the list. Thus, we reserved the peak with the highest local intensity in the range of three *m*/*z* and removed the other peaks near it.

### 4.4. Feature Extraction by a Two-Stage Alignment Method

In mass spectral data, peaks representing the same peptide may not be located at the same *m*/*z* in different spectra. Environmental factors, experimental procedures, and manual operation may cause small deviations in the measured peak positions. This phenomenon makes it difficult to use the extracted peaks as features for further analyses. We propose a two-stage alignment method to deal peak shifting problems.

In the first-stage, the spectrum was adjusted based on the housekeeping peak. We counted the frequency of peak occurrences in the *m*/*z* range from all spectra and chose the peak with the highest frequency as the housekeeping peak, which means that this peak was detected in almost all isolates [2]. For example, a study indicated that a peak at *m*/*z* 4429 was present in *Enterococcus* species [26]. The peak closest to the determined common peak in each spectrum was aligned with the position of the common peak, while the other peaks shifted by the same displacement. Each MS spectrum shifted according to the determined common peaks. If the MS spectrum did not contain a common peak, the distance between the closest peak and the common peak was calculated. Subsequently, all peaks shifted according to the distance and direction. A schematic illustration of the alignment to the common peak is shown in Supplementary Figure S1. In addition, Supplementary Table S9 provides the frequencies and percentages of the top five peak occurrences for different preprocessing methods in the training datasets. The *m*/*z* peak with rank 1 was determined as the housekeeping peak for each preprocessing method and species.

In the second stage, kernel density estimation (KDE) was used to define the locations of the peaks. KDE is a nonparametric approach for estimating the probability density function of random variables. After the first-stage alignment, the *m*/*z* range was split into several intervals in which no peaks from the same isolate would exist to ensure that the peaks in the intervals were independent. KDE was applied to estimate the distribution of peak positions for each interval:

$$\;f\left(x\right)=\frac{1}{nh}\overset{n}{\underset{i=1}{\sum }}K\left(\frac{x-x_{i}}{h}\right)$$

$$K\left(u\right)=\frac{1}{\sqrt{2\pi }}e^{-\frac{1}{2}u^{2}}$$

where *x* represents the random variable of the peaks, *x_i_* is the *m*/*z* value, *n* is the number of *m*/*z* values, *h* is the solve-the-equation bandwidth [27], and *K* is the kernel function. A Gaussian kernel function was selected for this study.

### 4.5. Alignment and Featurization

After estimating the distribution, we located the local extrema of the probability density function. Pairs of peaks with a gap smaller than the given threshold, set as 3, were regarded as the same peak. The peak at a lower frequency merged with the peak at a higher frequency. We also set a threshold for the local maxima. Values of local maxima that were not larger than 1% of all isolates were filtered out as noise. The local maxima represented the most frequent position at which the peaks appeared. These are the alignment benchmarks of peaks, and the range of the two adjacent local minima is the range of peak occurrences. Therefore, the peak in this range is aligned with the corresponding benchmark peak. The benchmark peaks are the input features used for further prediction. We ignored the peak intensity information. Only the presence or absence of benchmark peaks was recorded. If a benchmark peak appeared, we marked it as 1; otherwise, we marked it as 0.

The ensemble-based method was implemented to identify the importance of features [28]. Extremely randomized trees were used as ensemble members [29]. They were the first to build numerous tree-based models. Each tree was trained using all the training data. Second, a tree was constructed by randomly selecting a feature split. The importance of the features was determined based on the average impurity reduction over trees. We follow the rank of importance to form a subset of peaks. In this study, we built an extremely randomized tree model with 200 trees using the function “sklearn.ensemble.ExtraTreesClassifier” in the scikit-learn package [30].

### 4.6. Machine Learning Models

To identify the antibiotic resistance of each bacterium, we implemented four machine learning classification algorithms: logistic regression (LR), naïve Bayes (NB) classifier, random forest (RF), and support vector machine (SVM) using Python programming language (version 3.6.8).

The scikit-learn package [30] was used to build LR, NB, RF, and SVM models. More specifically, the “linear_model.LogisticRegression”, “naive_bayes.BernoulliNB”, “ensemble.RandomForestClassifier”, and “svm.SVC” were used to develop the LR, NB, RF, and SVM models, respectively. The primary objective of this study was to compare different preprocessing methods for MS. Almost all parameters used the default provided by the scikit-learn package [30], except some parameters that were used according to our previous research [4,22]. Specifically, the L2-norm was specified in the penalty and the maximum number of iterations was set to 1000 when LR models were constructed. In addition, to avoid overfitting, the number of trees in RF was set to 200. The kernel function in SVM was set as the radial basis function kernel. Detailed descriptions of these methods can be found in Supplementary Materials—Machine Learning Models.

### 4.7. Evaluation Metrics

In this study, stratified 10-fold cross-validation was performed to evaluate the performance of the models on the training datasets. Specifically, the training dataset was divided into ten equal-sized sub-datasets, which these maintained the percentage of data for each class. One sub-dataset was retained as the testing dataset for evaluating the model, which was built on the basis of the remaining sub-datasets. This procedure was repeated ten times. A different sub-dataset was selected as the testing dataset each time, with each sub-dataset used exactly once as the testing dataset. The 10 results obtained from these 10 sub-datasets were averaged to determine the overall performance of the models. The model with the best performance was then trained using all the training datasets and tested on the independent testing dataset. The metrics used to evaluate classification performance were sensitivity (SEN), specificity (SPE), accuracy (ACC), and area under the receiver operating characteristic curve (AUROC). The first three metrics are defined as follows:

$$\mathrm{ACC}=\frac{\mathrm{TP}+\mathrm{TN}}{\mathrm{TP}+\mathrm{TN}+\mathrm{FP}+\mathrm{FN}}$$

$$\mathrm{SEN}=\frac{\mathrm{TP}}{\mathrm{TP}+\mathrm{FN}}$$

$$\mathrm{SPE}=\frac{\mathrm{TN}}{\mathrm{TN}+\mathrm{FP}}$$

where TP is the true positive and denotes the number of resistant isolates that were correctly predicted by the model, TN is the true negative, denotes the number of susceptible isolates that were correctly predicted by the model, FP is the false positive, denotes the number of resistant isolates that were incorrectly predicted by the model, FN is the false negative and denotes the number of susceptible isolates that were incorrectly predicted by the model.

The binary classification model predicts a certain class based on a specified threshold. The receiver operating characteristic curve was generated by plotting the true positive rate, which is equal to the sensitivity, against the false positive rate, which is equal to 1 minus the specificity at all possible thresholds. The AUROC can be used as an indicator for comparing different classification models. In addition, the Youden index was chosen as the optimal cut-off point for classification [31].

## 5. Conclusions

Through proper feature selection, we selected the peaks that influenced antibiotic resistance and further analyzed the informative peaks for each bacterium. We found that some peaks could not be identified using certain individual preprocessing methods. Our ensemble method could yield more information owing to the integration of multiple preprocessing methods to detect more important peaks related to antibiotic resistance. Therefore, fewer peaks can be used to rapidly identify antibiotic resistance.

MALDI-TOF MS spectrum-based prediction of antibiotic resistance is a promising approach to provide rapid and accurate drug resistance information in a few days earlier than conventional tests. The early drug resistance information would significantly benefit patients’ survival. To generate robust predictions, however, several steps (e.g., Sample preparation, MALDI-TOF analysis, MS data preprocessing, and model training/validation) need to be standardized. Amid the issues, MS data preprocessing has not yet been well addressed. In the study, we compared several preprocessing methods and found that different preprocessing methods would significantly affect the predictive performance. The finding provides a new clue on the issue of standardizing MS data preprocessing. Appropriate MS data preprocessing method would be one of the keys to provide robust and reliable prediction of drug resistance to clinical physicians. Moreover, in terms of investigating the underlying mechanism of drug resistance, the set of discriminative peaks would be determined according to which MS data preprocessing method you used, which may direct our attention to different target proteins of drug resistance.

## Figures and Tables

**Table 1:** Numbers of features for different preprocessing methods and the corresponding performance after the feature selection was adopted based on RF.

Method	#Features	TP	TN	FP	FN	Sensitivity	Specificity	Accuracy	AUROC
(a) *A. baumannii,*									
FlexAnalysis	101	243.1 ± 9.60	177.7 ± 6.82	18.1 ± 6.57	38.6 ± 9.45	0.8630 ± 0.0337	0.9075 ± 0.0337	0.8812 ± 0.0154	0.9460 ± 0.0098
MALDIquant	117	234.3 ± 14.48	169.4 ± 8.15	26.4 ± 8.13	47.4 ± 14.23	0.8317 ± 0.0507	0.8652 ± 0.0415	0.8454 ± 0.0272	0.9218 ± 0.0152
CWT	88	250.2 ± 8.23	179.6 ± 4.03	16.2 ± 3.94	31.5 ± 8.11	0.8882 ± 0.0288	0.9173 ± 0.0201	0.9001 ± 0.0158	0.9564 ± 0.0076
Ensemble method	63	246.7 ± 6.96	185.0 ± 4.78	10.8 ± 4.96	35.0 ± 7.02	0.8758 ± 0.0249	0.9449 ± 0.0253	0.9041 ± 0.0137	0.9616 ± 0.0073
(b) *A. nosocomialis*									
FlexAnalysis	73	93.4 ± 5.89	92.3 ± 3.33	8.5 ± 3.44	15.5 ± 5.95	0.8577 ± 0.0545	0.9157 ± 0.0340	0.8855 ± 0.0223	0.9377 ± 0.0157
MALDIquant	50	90.0 ± 2.67	90.4 ± 3.41	10.4 ± 3.17	18.9 ± 2.60	0.8264 ± 0.0240	0.8968 ± 0.0318	0.8603 ± 0.0175	0.9186 ± 0.0176
CWT	32	92.7 ± 3.65	95.9 ± 1.97	4.9 ± 2.02	16.2 ± 3.82	0.8513 ± 0.0349	0.9514 ± 0.0201	0.8994 ± 0.0184	0.9417 ± 0.0160
Ensemble method	20	91.9 ± 4.33	96.5 ± 3.87	4.3 ± 3.68	17.0 ± 4.47	0.8439 ± 0.0409	0.9573 ± 0.0367	0.8984 ± 0.0204	0.9318 ± 0.0099
(c) *E. faecium*									
FlexAnalysis	86	222.5 ± 11.65	215.5 ± 16.35	67.2 ± 16.49	53.0 ± 11.58	0.8076 ± 0.0421	0.7623 ± 0.0582	0.7847 ± 0.0198	0.8474 ± 0.0222
MALDIquant	74	222.0 ± 6.88	206.2 ± 6.76	76.5 ± 7.06	53.5 ± 6.84	0.8058 ± 0.0248	0.7294 ± 0.0247	0.7671 ± 0.0163	0.8279 ± 0.0221
CWT	85	225.5 ± 12.66	212.1 ± 9.45	70.6 ± 9.22	50.0 ± 12.58	0.8185 ± 0.0457	0.7502 ± 0.0328	0.7839 ± 0.0172	0.8493 ± 0.0197
Ensemble method	62	219.2 ± 12.93	221.9 ± 8.61	60.8 ± 8.68	56.3 ± 12.93	0.7956 ± 0.0470	0.7849 ± 0.0307	0.7902 ± 0.0212	0.8565 ± 0.0203
(d) Group B *Streptococci*									
FlexAnalysis	33	152.5 ± 21.09	211.3 ± 25.54	75.7 ± 25.54	83.3 ± 20.91	0.6467 ± 0.0889	0.7362 ± 0.0890	0.6959 ± 0.0265	0.7442 ± 0.0272
MALDIquant	78	151.1 ± 14.98	211 ± 12.36	76.0 ± 12.36	84.7 ± 14.83	0.6408 ± 0.0631	0.7352 ± 0.0431	0.6926 ± 0.0152	0.7414 ± 0.0226
CWT	90	154.8 ± 14.86	211.9 ± 21.66	75.1 ± 21.66	81.0 ± 14.97	0.6565 ± 0.0633	0.7383 ± 0.0755	0.7014 ± 0.0191	0.7594 ± 0.0207
Ensemble method	39	155.2 ± 12.66	208.3 ± 19.12	78.7 ± 19.12	80.6 ± 12.85	0.6582 ± 0.0542	0.7258 ± 0.0666	0.6953 ± 0.0222	0.7463 ± 0.0252

Note. The means and standard deviations of the 10-fold cross-validation for each evaluation metrics were calculated. LR: CWT: Continuous Wavelet Transform; Logistic Regression; NB: Naïve Bayes; RF: Random Forest; SVM: Support Vector Machine; TP: True Positive; TN: True Negative; FP: False Positive; FN: False Negative; AUROC: Area Under the Receiver Operating Characteristic Curve.

**Table 2:** Performance of different species using ensemble method preprocessing and RF with selected features on the independent testing set.

Species	#Features	TP	TN	FP	FN	Sensitivity	Specificity	Accuracy	AUROC
*A. baumannii*	63	485	373	58	101	0.8276	0.8654	0.8437	0.9264
*A. nosocomialis*	20	73	88	4	12	0.8588	0.9565	0.9096	0.9391
*E. faecium*	62	927	767	229	234	0.7984	0.7701	0.7854	0.8569
Group B *Streptococci*	39	330	461	200	137	0.7066	0.6974	0.7012	0.7611

Note. LR: CWT: Continuous Wavelet Transform; Logistic Regression; NB: Naïve Bayes; RF: Random Forest; SVM: Support Vector Machine; TP: True Positive; TN: True Negative; FP: False Positive; FN: False Negative; AUROC: Area Under the Receiver Operating Characteristic Curve.

**Table 3:** Statistics of antibiotic resistance in training and independent testing datasets.

Species	Antibiotics	Resistant	Susceptible	Total
Training				
*A. nosocomialis*	CIP	1089	1008	2097
*A.baumannii*	CIP	2817	1958	4775
*E. faecium*	VA	2755	2827	5582
Group B *Streptococci*	CC	2358	2870	5228
Independent Testing				
*A. nosocomialis*	CIP	85	92	117
*A.baumannii*	CIP	586	431	1017
*E. faecium*	VA	1161	996	2157
Group B *Streptococci*	CC	467	661	1128

## Data Availability

The data presented in this study are available on request from the corresponding author. The data are not publicly available due to privacy.

## Supplementary Materials

The supporting information can be downloaded at: https://www.mdpi.com/article/10.3390/ijms24020998/s1.
