# Supplementary material for: Towards Accurate Identification of Antibiotic-Resistant Pathogens through the Ensemble of Multiple Preprocessing Methods Based on MALDI-TOF Spectra
Source: Int J Mol Sci. 2023 Jan 5;24(2):998. doi: 10.3390/ijms24020998 (PMC9865071; doi:10.3390/ijms24020998)
Supplement: Supplementary file 1 [file ijms-24-00998-s001.zip › ijms-2066154-supplementary.pdf]

Towards Accurate Identification of Antibiotic-Resistant Pathogens through the Ensemble of Multiple Preprocessing Methods Based on MALDI-TOF Spectra

Chia-Ru Chung[1,2,†], Hsin-Yao Wang[3,4,†], Po-Han Chou[5], Li-Ching Wu[6], Jang-Jih Lu[3,7,8,9], Jorng-Tzong Horng[1,10,*], and Tzong-Yi Lee[11,12,*]

[1]Kobilka Institute of Innovative Drug Discovery, School of Medicine, The Chinese University of Hong Kong, Shenzhen 518172, China

[2]School of Life Sciences, University of Science and Technology of China, Hefei 230026, China

[3]Department of Laboratory Medicine, Chang Gung Memorial Hospital at Linkou, Taoyuan 333423, Taiwan

[4]Ph.D. Program in Biomedical Engineering, Chang Gung University, Taoyuan 333323, Taiwan

[5]Department of Computer Science and Information Engineering, National Central University, Taoyuan 320317, Taiwan

[6]Department of Biomedical Sciences and Engineering, National Central University, Taoyuan 320317, Taiwan

[7]Research Center for Emerging Viral Infections, Chang Gung University, Taoyuan 333323, Taiwan

[8]College of Medicine, Chang Gung University, Taoyuan 333323, Taiwan

[9]Department of Medical Biotechnology and Laboratory Science, Chang Gung University, Taoyuan 333323, Taiwan

[10]Department of Bioinformatics and Medical Engineering, Asia University, Taichung 41354, Taiwan

[11]Warshel Institute for Computational Biology, School of Life and Health Sciences, The Chinese University of Hong Kong, Shenzhen 518172, China

[12] Institute of Bioinformatics and Systems Biology, National Yang Ming Chiao Tung University, Hsinchu 300093, Taiwan

[†]These authors contributed equally to this work.

[*]To whom correspondence should be addressed: JT Horng: horng@db.csie.ncu.edu.tw and TY Lee: francislee0215@gmail.com

Supplementary Materials

Machine Learning Models

To identify the antibiotic resistance of each bacterium, we implemented four machine learning classification algorithms: logistic regression (LR), naïve Bayes (NB) classifier, random forests (RFs), and support vector machine (SVM) using the Python programming language (version 3.6.8).

LR is a statistical model that uses logistic function to model binary dependent variables. In this study, the two dependent variables were non-susceptible and susceptible. Non-susceptible isolates were labeled 1, and susceptible isolates were labeled 0. Benchmark peaks were identified as predictors. The probability of labels was calculated using the logistic function of the linear combination of multiple predictors.

$$P(y)=\frac{1}{1+e^{-(a_0+\sum_{i=1}^{n} a_i x_i)}}$$

where y is the label, P is the probability of the label, and x_i is the predictors. We used the "sklearn.linear_model.LogisticRegression" function in the scikit-learn package to construct a LR model [1]. The L2-norm was specified in the penalty, and the maximum number of iterations was set to 1000.

NB classifiers are probabilistic classifiers based on Bayes' theorem, with the assumptions of naïve independence between features. Bayes' theorem states that the probability of an event, representing susceptible or not, is based on prior knowledge. Bayes' theorem is as follows:

$$P(y|x_1,\dots,x_n)=\frac{P(y)P(x_1,..,x_n|y)}{P(x_1,\dots,x_n)}$$

$$P(x_1,\dots,x_n|y)=\prod_{i=1}^{n} p_y^{x_i}\left(1-p_y\right)^{(1-x_i)}$$

where $P(y|x_1,\dots,x_n)$ is the posterior probability of an event, $P(y)$ and $P(x_1,\dots,x_n)$ are the prior probabilities of an event and features, and $P(x_1,\dots,x_n|y)$ is the likelihood. The likelihood follows a multivariate Bernoulli distribution. In this study, we used the "sklearn.naive_bayes.BernoulliNB" function in the scikit-learn package [1].

RFs are an ensemble learning method that involves constructing several decision trees to output the class based on the classes of individual trees. This method uses bootstrap aggregating to generate multiple sub-isolate sets by sampling from the dataset

randomly and with replacement. Each sub-isolate set was input into the decision tree as a training set. To avoid the correlation of trees caused by bootstrap sampling, the features of each decision tree would be a random subset of the total features. A decision tree is a tree-structure model constructed using the top-down divide-and-conquer strategy. The internal node of a tree represents a feature. The isolates are divided into partitions based on selected features recursively. In this study, the Gini index was used to measure the impurities in the partition. The formula for the Gini index is

$$\mathrm{Gini}(D)=\sum_{i=1}^{n} p_i^2,$$

where D is the dataset, n is the number of classes in D, and p_i is the probability of class i. If dataset D is split on feature A into subsets D_i, the Gini index gives the split on feature A as

$$\mathrm{Gini}_{\mathrm{A}}(D)=\sum_{i}\frac{|D_i|}{|D|}\mathrm{Gini}(D_i)$$

Furthermore, the reduction of impurity is:

$$\Delta\mathrm{Gini(A)}=\mathrm{Gini(D)}-\mathrm{Gini}_{\mathrm{A}}(\mathrm{D}_i)$$

The feature providing maximum reduction in impurity is selected as the split attribution at a node during the generation stage. In this study, the function "sklearn.ensemble.RandomForestClassifier" in the scikit-learn package was applied to establish an RF model with 200 trees [1].

SVMs are popular supervised machine learning classifications for both linear and nonlinear data. They use appropriate nonlinear mapping to transform training data into a high-dimensional space and construct a hyperplane to separate the two classes. The hyperplane can be written as follows:

$$W*X+b=0$$

where $W = (w_1, w_2, \ldots, w_n)$ is the weight vector, and b is the bias. Hyperplanes are determined by support vectors chosen from the training data. The best hyperplane had the largest margin between the support vectors. The SVM model in this study was implemented using the "sklearn.svm.SVC" function in the scikit-learn package [1]. The kernel function was set as the radial basis function kernel, and the other parameters were as default.

Supplementary Figures

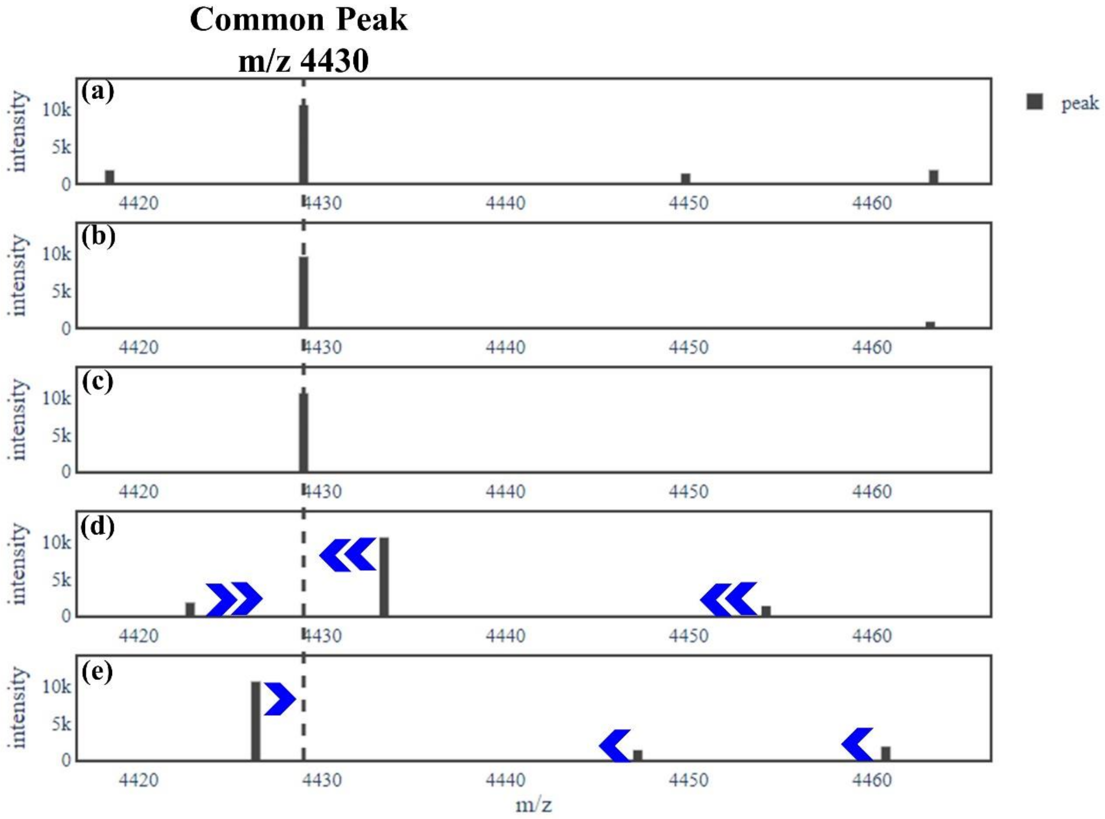

Supplementary Figure S1. Schematic illustration of aligning to common peak when the m/z 4430 was the common peak. If a MS spectrum contains a peak with m/z 4430, then it did not need to align, such as (a), (b), and (c); otherwise, the distance between the nearest m/z 4430 was calculated, and then all peaks was shifted based on the distance, such as (d), and (e).

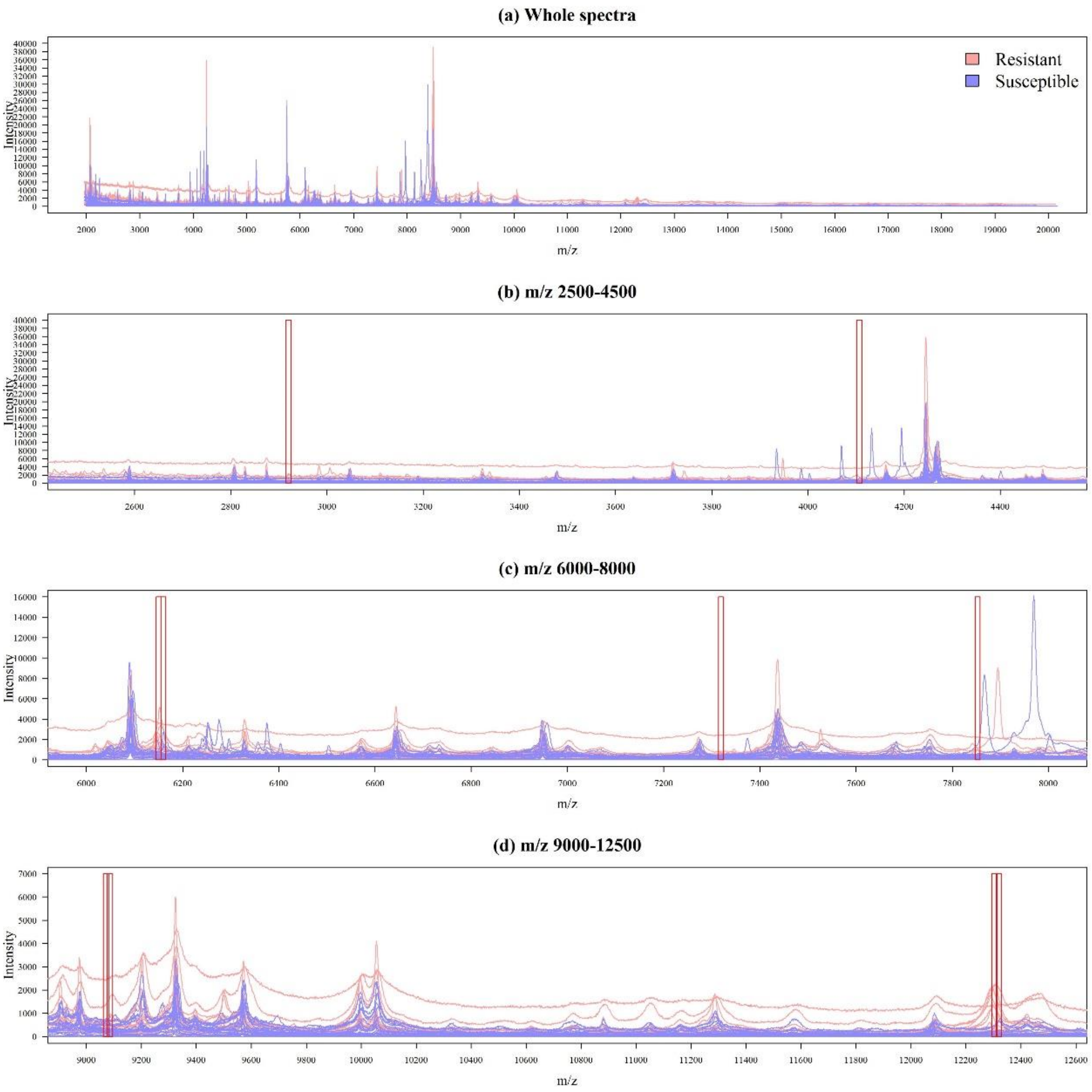

Supplementary Figure S2. MALDI-TOF MS profiles of *A. baumannii* isolates and the peaks which highly affected the performance of prediction model. MALDI-TOF MS spectra of *A. baumannii* isolates are depicted as (a) whole spectrum (m/z 2000-20000), (b) m/z 2500-4500, (c) m/z 6000-8000, and (d) m/z 9000-125000. The top 10 peaks which highly affected the performance of prediction model are labeled with red rectangles.

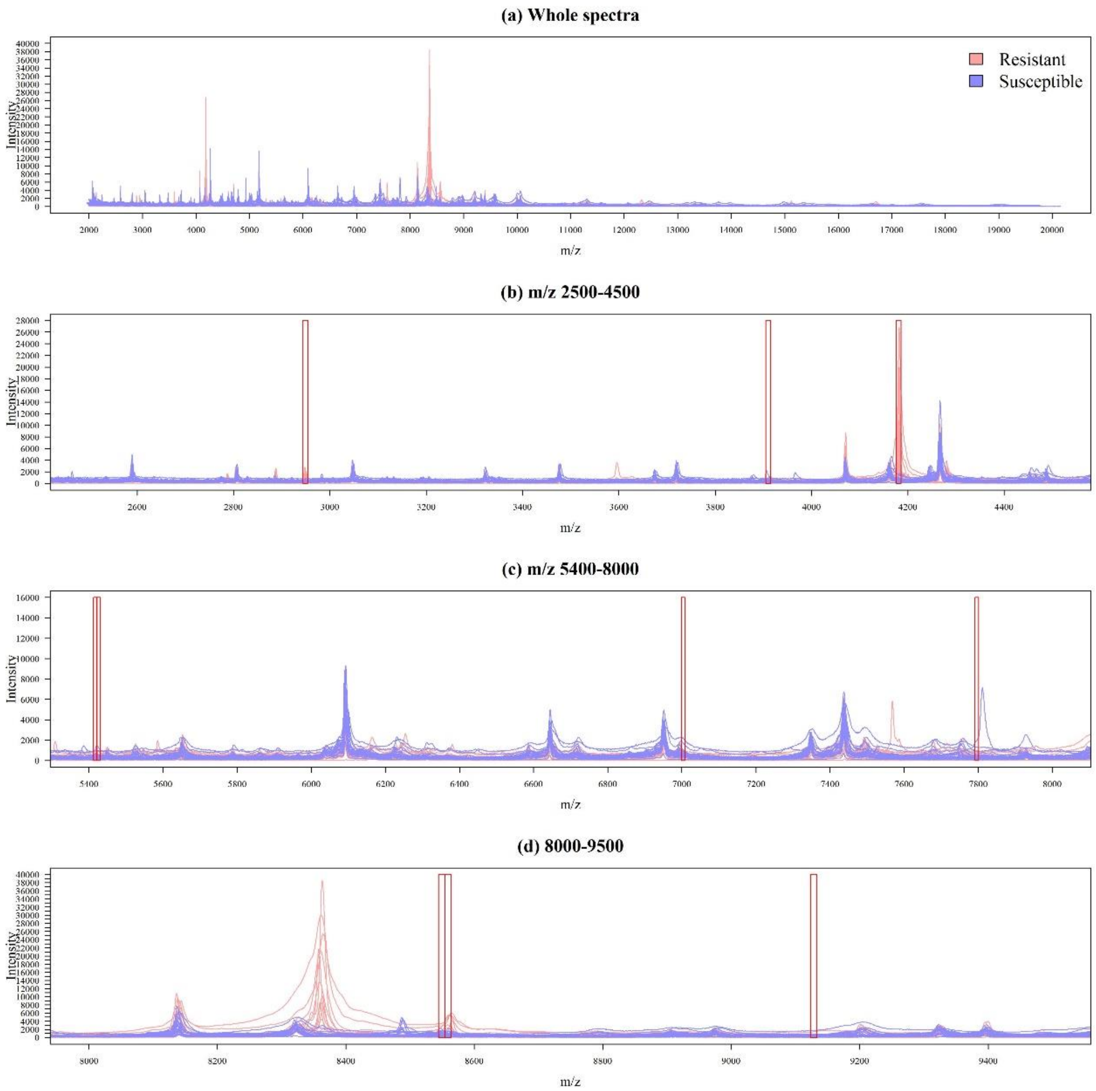

Supplementary Figure S3. MALDI-TOF MS profiles of *A. nosocomialis* isolates and the peaks which highly affected the performance of prediction model. MALDI-TOF MS spectra of *A. nosocomialis* isolates are depicted as (a) whole spectrum (m/z 2000-20000), (b) m/z 2500-4500, (c) m/z 5400-8000, and (d) m/z 8000-9500. The top 10 peaks which highly affected the performance of prediction model are labeled with red rectangles.

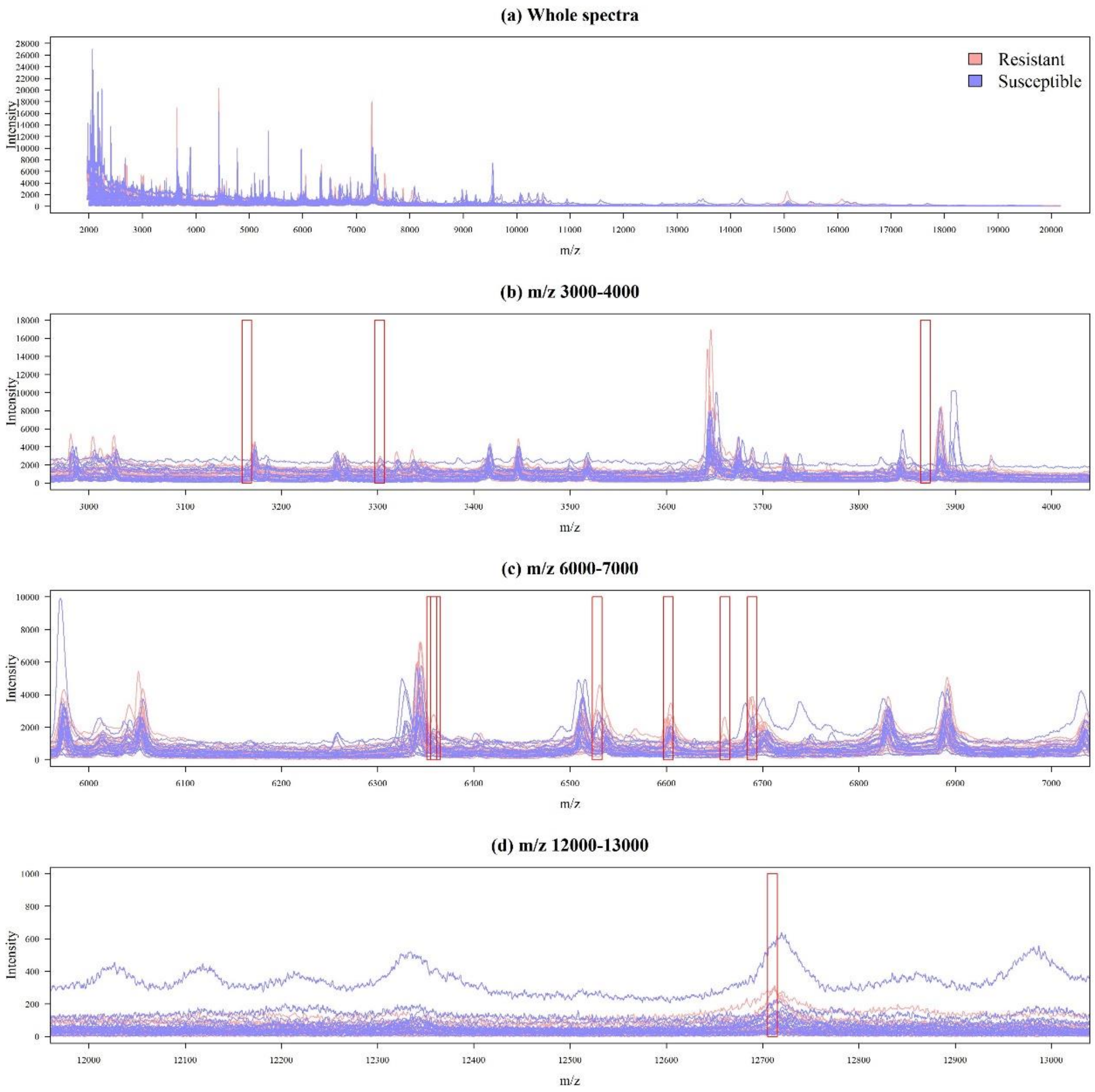

Supplementary Figure S4. MALDI-TOF MS profiles of *E. faecium* isolates and the peaks which highly affected the performance of prediction model. MALDI-TOF MS spectra of *E. faecium* isolates are depicted as (a) whole spectrum (m/z 2000-20000), (b) m/z 3000-4000, (c) m/z 6000-7000, and (d) m/z 12000-13000. The top 10 peaks which highly affected the performance of prediction model are labeled with red rectangles.

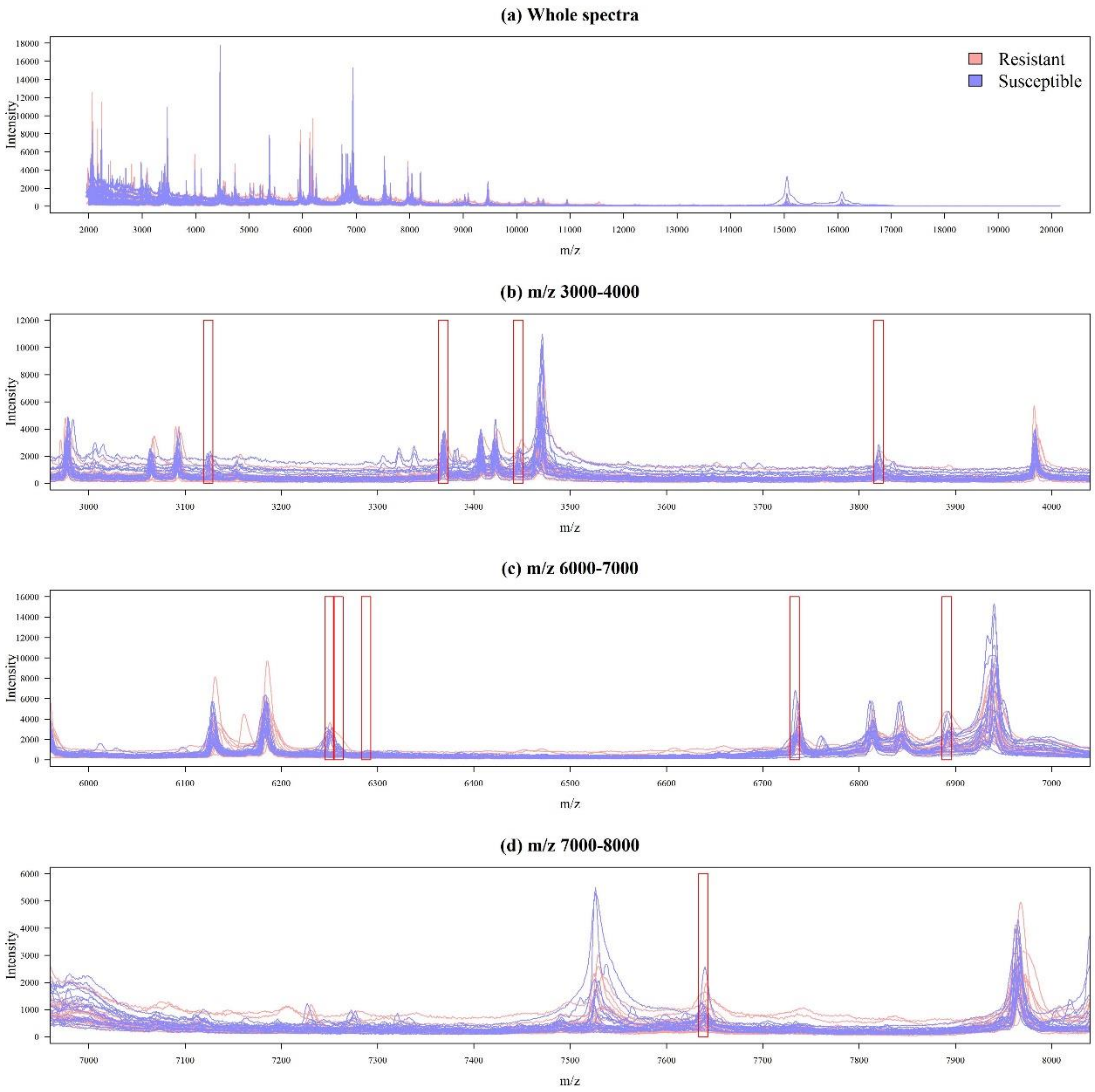

Supplementary Figure S5. MALDI-TOF MS profiles of *Group B Streptococci* isolates and the peaks which highly affected the performance of prediction model. MALDI-TOF MS spectra of *Group B Streptococci* isolates are depicted as (a) whole spectrum (m/z 2000-20000), (b) m/z 3000-4000, (c) m/z 6000-7000, and (d) m/z 7000-8000. The top 10 peaks which highly affected the performance of prediction model are labeled with red rectangles.

Supplementary Tables

Supplementary Table S1. 10-fold cross validation performance of *A. baumannii* on training datasets.

Method	Model	TP	TN	FP	FN	Sensitivity	Specificity	Accuracy	AUROC
Flexanalysis	LR	234.4±7.06	169.1±6.01	26.7±6.15	47.3±6.99	0.8321±0.0248	0.8637±0.0313	0.8450±0.0218	0.9179±0.0172
	NB	207.4±16.11	150.9±15.04	44.9±15.17	74.3±15.97	0.7362±0.0568	0.7707±0.0773	0.7504±0.0248	0.8163±0.0236
	RF	242.4±8.40	176.2±8.60	19.6±8.64	39.3±8.47	0.8605±0.0301	0.8999±0.0441	0.8766±0.0128	0.9425±0.0098
	SVM	238.6±9.36	173.4±6.77	22.4±6.60	43.1±9.46	0.8470±0.0335	0.8856±0.0338	0.8628±0.0166	0.9311±0.0128
MALDIquant	LR	224.3±9.89	168.5±3.41	27.3±3.47	57.4±9.79	0.7962±0.0348	0.8606±0.0176	0.8226±0.0219	0.9013±0.0188
	NB	192.4±27.08	150.3±19.79	45.5±19.81	89.3±27.23	0.6830±0.0965	0.7676±0.1011	0.7177±0.0290	0.7884±0.0245
	RF	230.4±9.63	170.8±7.38	25.0±7.38	51.3±9.44	0.8179±0.0336	0.8723±0.0377	0.8402±0.0223	0.9192±0.0151
	SVM	236.7±11.21	163.1±9.15	32.7±9.02	45±11.24	0.8403±0.0399	0.8330±0.0461	0.8373±0.0176	0.9064±0.0149
CWT	LR	232.5±10.47	179.2±4.98	16.6±5.15	49.2±10.15	0.8253±0.0363	0.9152±0.0262	0.8622±0.0156	0.9336±0.0104
	NB	200.1±13.58	171.2±11.39	24.6±11.2	81.6±13.62	0.7103±0.0483	0.8743±0.0574	0.7776±0.0172	0.8521±0.0148
	RF	242.4±9.23	183.3±3.50	12.5±3.66	39.3±9.29	0.8605±0.0329	0.9362±0.0186	0.8915±0.0159	0.9543±0.0069
	SVM	245±9.01	180.6±6.77	15.2±6.70	36.7±9.02	0.8697±0.0320	0.9224±0.0342	0.8913±0.0140	0.9470±0.0081
Ensemble method	LR	240.0±10.02	174.5±5.60	21.3±5.50	41.7±9.91	0.8520±0.0352	0.8912±0.0281	0.8681±0.0161	0.9366±0.0093
	NB	195.8±18.21	172.7±12.24	23.1±12.20	85.9±18.36	0.6951±0.0650	0.8820±0.0623	0.7717±0.0231	0.8462±0.0147
	RF	247.5±6.13	183.6±4.95	12.2±4.98	34.2±6.03	0.8786±0.0215	0.9377±0.0254	0.9028±0.0118	0.9617±0.0068
	SVM	247.8±7.67	182.1±3.84	13.7±3.74	33.9±7.8	0.8797±0.0276	0.9300±0.0191	0.9003±0.0155	0.9563±0.0089

Note. LR: CWT: Continuous Wavelet Transform; Logistic Regression; NB: Naïve Bayes; RF: Random Forest; SVM: Support Vector Machine; TP: True Positive; TN: True Negative; FP: False Positive; FN: False Negative; AUROC: Area Under the Receiver Operating Characteristic Curve.

Supplementary Table S2. 10-fold cross validation10-fold cross validation performance of *A. nosocomialis* on training datasets.

Method	Model	TP	TN	FP	FN	Sensitivity	Specificity	Accuracy	AUROC
Flexanalysis	LR	84.4±6.33	89.5±6.20	11.3±6.13	24.5±6.29	0.7750±0.0578	0.8879±0.0610	0.8293±0.0366	0.8929±0.0302
	NB	81.5±6.17	87.8±6.88	13.0±6.90	27.4±6.15	0.7484±0.0565	0.8710±0.0682	0.8073±0.0182	0.8669±0.0244
	RF	89.7±3.16	92.1±3.25	8.7±2.87	19.2±3.19	0.8237±0.0292	0.9136±0.0289	0.8669±0.0237	0.9244±0.0165
	SVM	86.8±5.35	93.3±2.50	7.5±2.64	22.1±5.45	0.7971±0.0498	0.9256±0.0260	0.8588±0.0195	0.9081±0.0168
MALDIquant	LR	85.6±5.32	88.3±7.33	12.5±7.32	23.3±5.36	0.7861±0.0490	0.8760±0.0726	0.8293±0.0321	0.8881±0.0255
	NB	79.9±8.06	90.6±4.81	10.2±4.76	29.0±8.06	0.7337±0.0739	0.8988±0.0471	0.8131±0.0288	0.8722±0.0255
	RF	87.2±4.94	91.3±3.23	9.5±3.21	21.7±4.83	0.8007±0.0446	0.9058±0.0318	0.8512±0.0231	0.9163±0.0183
	SVM	85.4±5.66	90.3±4.50	10.5±4.20	23.5±5.64	0.7842±0.0518	0.8957±0.0421	0.8379±0.0239	0.8994±0.0184
CWT	LR	89.4±5.56	90.8±5.81	10.0±5.58	19.5±5.76	0.8210±0.0527	0.9007±0.0556	0.8593±0.0223	0.9177±0.0142
	NB	85.5±6.40	93.1±4.98	7.7±4.74	23.4±6.59	0.7852±0.0602	0.9235±0.0473	0.8517±0.0232	0.9084±0.0219
	RF	93.8±2.74	93.0±3.27	7.8±2.97	15.1±2.85	0.8614±0.0260	0.9225±0.0298	0.8908±0.0183	0.9387±0.0150
	SVM	89.9±4.53	94.2±4.02	6.6±3.81	19.0±4.50	0.8255±0.0413	0.9345±0.0379	0.8779±0.0155	0.9290±0.0122
Ensemble method	LR	91.8±4.32	90.6±6.00	10.2±5.87	17.1±4.38	0.8430±0.0401	0.8987±0.0586	0.8698±0.0193	0.9311±0.0114
	NB	84.7±4.76	92.3±5.06	8.5±5.02	24.2±4.83	0.7778±0.0441	0.9157±0.0497	0.8440±0.0236	0.9011±0.0234
	RF	93.4±3.24	94.6±2.41	6.2±2.44	15.5±3.37	0.8577±0.0308	0.9385±0.0241	0.8965±0.0145	0.9444±0.0125
	SVM	92.6±3.37	95.2±2.15	5.6±2.01	16.3±3.53	0.8504±0.0322	0.9444±0.0200	0.8956±0.0124	0.9373±0.0115

Note. LR: CWT: Continuous Wavelet Transform; Logistic Regression; NB: Naïve Bayes; RF: Random Forest; SVM: Support Vector Machine; TP: True Positive; TN: True Negative; FP: False Positive; FN: False Negative; AUROC: Area Under the Receiver Operating Characteristic Curve.

Supplementary Table S3. 10-fold cross validation10-fold cross validation performance of *E. faecium* on training datasets.

Method	Model	TP	TN	FP	FN	Sensitivity	Specificity	Accuracy	AUROC
Flexanalysis	LR	222.0±15.11	200.7±18.07	82.0±18.17	53.5±14.89	0.8058±0.0543	0.7100±0.0641	0.7573±0.0159	0.8218±0.0197
	NB	202.3±12.67	205.1±14.92	77.6±15.25	73.2±12.81	0.7343±0.0463	0.7256±0.0536	0.7298±0.0211	0.7876±0.0304
	RF	216.3±13.06	218.5±11.35	64.2±11.51	59.2±12.98	0.7851±0.0471	0.7729±0.0406	0.7789±0.0183	0.8462±0.0203
	SVM	219.7±11.39	211.2±14.84	71.5±15.06	55.8±11.47	0.7975±0.0416	0.7471±0.0531	0.7719±0.0144	0.8369±0.0172
MALDIquant	LR	214.7±14.10	201.4±13.03	81.3±13.20	60.8±14.02	0.7793±0.0509	0.7124±0.0465	0.7454±0.021	0.8085±0.0219
	NB	215.4±19.70	169.2±31.12	113.5±31.28	60.1±20.00	0.7819±0.0723	0.5986±0.1104	0.6890±0.0308	0.7429±0.0434
	RF	215.4±11.69	211.4±20.71	71.3±20.76	60.1±11.84	0.7819±0.0429	0.7478±0.0733	0.7646±0.0232	0.8321±0.0213
	SVM	209.5±16.10	214.7±17.19	68.0±17.23	66.0±15.90	0.7604±0.0579	0.7595±0.0609	0.7599±0.0176	0.8237±0.0226
CWT	LR	212.8±17.02	204.3±14.89	78.4±14.98	62.7±17.02	0.7724±0.0617	0.7227±0.0529	0.7472±0.0154	0.8095±0.0141
	NB	195.2±17.46	210.5±19.52	72.2±19.67	80.3±17.59	0.7086±0.0637	0.7446±0.0695	0.7268±0.0250	0.7871±0.0305
	RF	224.3±15.24	209.0±14.97	73.7±15.16	51.2±15.13	0.8141±0.0550	0.7393±0.0535	0.7762±0.0193	0.8433±0.0199
	SVM	220.2±13.29	208.1±17.56	74.6±17.56	55.3±13.44	0.7993±0.0486	0.7361±0.0621	0.7673±0.0157	0.8327±0.0180
Ensemble method	LR	217.7±14.52	201.4±16.69	81.3±16.72	57.8±14.30	0.7902±0.0521	0.7124±0.0590	0.7508±0.0170	0.8185±0.0206
	NB	207.6±17.86	200.3±18.68	82.4±18.83	67.9±17.83	0.7535±0.0647	0.7086±0.0664	0.7307±0.0263	0.7916±0.0317
	RF	227.7±14.44	212.7±13.57	70.0±13.66	47.8±14.31	0.8265±0.0519	0.7524±0.0482	0.7890±0.0238	0.8537±0.0232
	SVM	228.5±14.57	206.4±17.10	76.3±17.05	47.0±14.64	0.8294±0.0530	0.7301±0.0604	0.7791±0.0221	0.8462±0.0199

Note. LR: CWT: Continuous Wavelet Transform; Logistic Regression; NB: Naïve Bayes; RF: Random Forest; SVM: Support Vector Machine; TP: True Positive; TN: True Negative; FP: False Positive; FN: False Negative; AUROC: Area Under the Receiver Operating Characteristic Curve.

Supplementary Table S4. 10-fold cross validation10-fold cross validation performance of *Group B Streptococci* on training datasets.

Method	Model	TP	TN	FP	FN	Sensitivity	Specificity	Accuracy	AUROC
Flexanalysis	LR	157.2±22.75	201.8±16.14	85.2±16.14	78.6±22.72	0.6667±0.0963	0.7031±0.0562	0.6867±0.0237	0.7337±0.0303
	NB	166.9±14.07	185.6±14.37	101.4±14.37	68.9±14.00	0.7078±0.0594	0.6467±0.0501	0.6742±0.0226	0.7149±0.0268
	RF	159.5±21.79	206.2±23.28	80.8±23.28	76.3±21.62	0.6764±0.0919	0.7185±0.0811	0.6995±0.0205	0.7600±0.0264
	SVM	151.1±15.37	218.8±17.43	68.2±17.43	84.7±15.44	0.6408±0.0653	0.7624±0.0607	0.7075±0.0226	0.7516±0.0276
MALDIquant	LR	156.9±15.44	193.8±21.59	93.2±21.59	78.9±15.47	0.6654±0.0657	0.6753±0.0752	0.6708±0.0232	0.7219±0.0258
	NB	174.5±18.08	176.3±21.95	110.7±21.95	61.3±18.10	0.7400±0.0767	0.6143±0.0765	0.6710±0.0249	0.7207±0.0307
	RF	162.2±18.81	200.2±21.35	86.8±21.35	73.6±18.58	0.6878±0.0792	0.6976±0.0744	0.6932±0.0214	0.7432±0.0261
	SVM	153.1±11.72	209.1±12.73	77.9±12.73	82.7±11.42	0.6492±0.0489	0.7286±0.0444	0.6928±0.0227	0.7322±0.0228
CWT	LR	149.7±19.32	204.3±23.00	82.7±23.00	86.1±19.32	0.6349±0.0820	0.7118±0.0801	0.6771±0.0185	0.7221±0.0246
	NB	161.4±17.47	193.2±24.03	93.8±24.03	74.4±17.60	0.6845±0.0744	0.6732±0.0837	0.6783±0.0205	0.7322±0.0258
	RF	163.0±10.72	200.4±14.48	86.6±14.48	72.8±10.64	0.6913±0.0452	0.6983±0.0504	0.6951±0.0201	0.7569±0.0223
	SVM	151.4±11.75	213.0±16.99	74.0±16.99	84.4±11.66	0.6421±0.0496	0.7422±0.0592	0.6970±0.0227	0.7401±0.0207
Ensemble method	LR	156.1±21.39	200.4±25.21	86.6±25.21	79.7±21.54	0.6621±0.0912	0.6983±0.0878	0.6819±0.0172	0.7243±0.0219
	NB	168.7±15.66	183.9±20.40	103.1±20.40	67.1±15.50	0.7154±0.0660	0.6408±0.0711	0.6744±0.0240	0.7229±0.0256
	RF	165.4±17.88	197.1±25.71	89.9±25.71	70.4±18.01	0.7015±0.0762	0.6868±0.0896	0.6934±0.0286	0.7552±0.0269
	SVM	155.6±18.25	212.4±19.87	74.6±19.87	80.2±18.33	0.6599±0.0776	0.7401±0.0692	0.7039±0.0211	0.7498±0.0220

Note. LR: CWT: Continuous Wavelet Transform; Logistic Regression; NB: Naïve Bayes; RF: Random Forest; SVM: Support Vector Machine; TP: True Positive; TN: True Negative; FP: False Positive; FN: False Negative; AUROC: Area Under the Receiver Operating Characteristic Curve.

Supplementary Table S5. Occurrence frequency of important peaks for *A. nosocomialis.*

Rank	Peak	Combined method		FlexAnalysis		MALDIquant		CWT	
		R(%)	S(%)	R(%)	S(%)	R(%)	S(%)	R(%)	S(%)
1	2949	53.4	3.5	45.0	1.3	43.9	1.5	48.0	3.5
2	4181	70.9	22.6	69.4	19.9	59.3	9.4	62.7	14.9
3	8549	8.7	52.2	8.4	47.5	6.1	44.4	5.5	47.3
4	3910	40.4	1.6	21.7	0.5	26.7	0.6	35.0	1.2
5	8559	74.2	29.6	65.7	23.4	62.8	21.8	68.0	26.9
6	5417	8.7	39.7	2.4	7.5	2.5	10.3	8.3	38.4
7	7003	17.8	6.2	15.3	4.4	4.6	0.3	9.7	2.2
8	7796	23.6	1.7	3.9	1.6	1.7	0.2	22.1	0.2
9	9128	23.3	0.4	15.8	0.2	4.7	0.0	17.5	0.3
10	5425	27.8	2.1	6.1	0.4	7.3	0.5	27.2	2.1

Supplementary Table S6. Occurrence frequency of important peaks for *A. baumannii.*

Rank	Peak	Combined method		FlexAnalysis		MALDIquant		CWT	
		R(%)	S(%)	R(%)	S(%)	R(%)	S(%)	R(%)	S(%)
1	6160	3.7	47.6	2.8	41.3	1.8	32.1	2.8	40.1
2	7853	45.1	8.9	9.1	5.7	6.4	3.7	42.5	7.8
3	9072	2.7	33.0	1.0	16.4	0.2	6.2	2.3	28.2
4	12320	2.1	30.7	1.5	24.4	0.4	12.0	1.3	24.0
5	6150	49.8	15.5	40.4	12.8	26.1	2.2	38.8	9.4
6	7319	30.8	2.9	3.7	1.0	0.5	0.2	29.1	2.1
7	9086	36.7	5.7	18.0	2.6	7.3	1.2	31.7	4.7
8	2920	44.8	19.0	20.1	4.6	29.9	9.6	38.7	16.6
9	12303	19.8	0.9	14.7	0.8	7.4	0.4	16.0	0.4
10	4107	1.0	17.4	0.5	10.9	0.4	8.2	1.1	14.9

Supplementary Table S7. Occurrence frequency of important peaks for *E. faecium*.

Rank	Peak	Combined method		FlexAnalysis		MALDIquant		CWT	
		R(%)	S(%)	R(%)	S(%)	R(%)	S(%)	R(%)	S(%)
1	6689	48.5	14.8	48.2	13.7	31.5	10.8	22.2	10.0
2	6356	49.9	22.9	49.0	22.3	11.1	4.3	5.8	2.4
3	6602	93.6	66.3	89.6	60.0	89.8	60.3	93.1	65.9
4	3302	92.7	67.8	70.5	40.4	88.2	61.6	91.6	67.4
5	6360	6.6	25.1	6.2	21.5	0.9	9.2	3.0	11.4
6	6661	24.9	7.2	18.5	4.6	17.5	4.2	15.8	5.2
7	6528	21.1	6.4	20.8	6.3	19.6	5.4	18.8	5.5
8	3869	5.7	21.8	4.5	14.6	1.2	8.8	1.8	12.5
9	12710	38.0	19.0	19.3	7.4	0.4	0.3	37.1	18.5
10	3164	0.4	16.9	0.3	16.6	0.4	16.5	0.3	16.7

Supplementary Table S8. Occurrence frequency of important peaks for Group B *Streptococci*.

Rank	Peak	Combined method		FlexAnalysis		MALDIquant		CWT	
		R(%)	S(%)	R(%)	S(%)	R(%)	S(%)	R(%)	S(%)
1	6250	33.6	67.4	32.8	67.0	32.3	66.2	33.0	67.0
2	3124	40.7	70.7	32.5	66.3	37.8	69.3	38.2	69.5
3	3368	96.9	81.9	96.3	81.4	96.3	81.3	96.9	81.7
4	7638	76.0	90.5	72.1	85.1	61.0	68.9	73.5	87.4
5	6733	96.0	82.1	95.9	82.0	95.3	79.8	95.7	80.8
6	3820	82.3	95.3	67.1	77.0	79.5	91.0	79.6	94.5
7	6288	19.3	41.6	18.5	41.1	6.9	15.3	7.8	16.8
8	6891	71.6	49.0	70.7	47.5	61.2	31.7	64.2	35.6
9	6259	37.7	17.7	32.4	15.2	12.5	6.4	25.1	11.7
10	3446	72.6	52.0	71.1	49.5	65.0	35.4	65.6	35.2

Supplementary Table S9. Frequency (Percentage) of the top five m/z peaks occurrences for different preprocessing methods on training datasets.

	**FlexAnalysis**		**MALDIquant**		**CWT**		**Ensemble method**	
	**m/z**	**Frequency (Percentage)**	**m/z**	**Frequency (Percentage)**	**m/z**	**Frequency (Percentage)**	**m/z**	**Frequency (Percentage)**
**Rank**	(a) *A. baumannii,*							
**1**	3719	1421(29.76)	3719	1439(30.14)	3476	1393(29.17)	3476	1468(30.74)
**2**	4267	1393(29.17)	3476	1289(26.99)	3047	1387(29.05)	3719	1454(30.45)
**3**	3476	1342(28.1)	5177	1286(26.93)	4267	1371(28.71)	3322	1426(29.86)
**4**	5177	1293(27.08)	3322	1273(26.66)	3719	1290(27.02)	4267	1396(29.24)
**5**	3046	1270(26.6)	4267	1260(26.39)	4663	1248(26.14)	3046	1342(28.1)
	(b) *A. nosocomialis*							
**1**	3719	703(33.52)	3719	703(33.52)	3047	669(31.9)	3719	717(34.19)
**2**	3476	618(29.47)	3476	584(27.85)	3476	651(31.04)	3476	668(31.86)
**3**	4267	611(29.14)	3046	578(27.56)	3719	643(30.66)	3322	621(29.61)
**4**	4162	586(27.94)	4267	578(27.56)	4071	623(29.71)	3675	619(29.52)
**5**	3046	574(27.37)	4070	566(26.99)	3675	622(29.66)	4267	613(29.23)
	(c) *E. faecium*							
**1**	3446	1838(32.93)	3446	1860(33.32)	3446	1866(33.43)	3446	1912(34.25)
**2**	3674	1836(32.89)	3415	1841(32.98)	3416	1846(33.07)	3415	1853(33.2)
**3**	3415	1754(31.42)	3172	1769(31.69)	3518	1799(32.23)	3674	1848(33.11)
**4**	3172	1729(30.97)	3518	1766(31.64)	3028	1694(30.35)	3518	1839(32.95)
**5**	3027	1637(29.33)	3674	1752(31.39)	3172	1672(29.95)	3172	1749(31.33)
	(d) Group B *Streptococci*							
**1**	3422	1873(35.83)	3407	1864(35.65)	3408	1726(33.01)	3422	1877(35.9)
**2**	3407	1866(35.69)	3422	1806(34.54)	3093	1699(32.5)	3407	1871(35.79)
**3**	3092	1790(34.24)	3092	1773(33.91)	4102	1691(32.35)	3092	1796(34.35)
**4**	3065	1736(33.21)	3065	1754(33.55)	3369	1659(31.73)	3065	1778(34.01)
**5**	2978	1723(32.96)	3983	1688(32.29)	3407	1644(31.45)	2978	1728(33.05)

References

1. Pedregosa F, Varoquaux G, Gramfort A et al. Scikit-learn: Machine Learning in Python, Journal of machine learning research 2011;12:2825-2830.
